# Soluble PD-L1 Is an Independent Prognostic Factor in Clear Cell Renal Cell Carcinoma

**DOI:** 10.3390/cancers13040667

**Published:** 2021-02-07

**Authors:** Gorka Larrinaga, Jon Danel Solano-Iturri, Peio Errarte, Miguel Unda, Ana Loizaga-Iriarte, Amparo Pérez-Fernández, Enrique Echevarría, Aintzane Asumendi, Claudia Manini, Javier C. Angulo, José I. López

**Affiliations:** 1 Department of Nursing, Faculty of Medicine and Nursing, University of the Basque Country (UPV/EHU), 48940 Leioa, Spain; 2 Department of Physiology, Faculty of Medicine and Nursing, University of the Basque Country (UPV/EHU), 48940 Leioa, Spain; peio@onenameds.com (P.E.); enrique.etxebarria@ehu.eus (E.E.); 3 BioCruces-Bizkaia Health Research Institute, 48903 Barakaldo, Spain; jondanel.solanoiturri@osakidetza.eus (J.D.S.-I.); joseignacio.lopez@osakidetza.eus (J.I.L.); 4 Department of Pathology, Donostia University Hospital, 20014 San Sebastian-Donostia, Spain; 5 Department of Medical-Surgical Specialities, Faculty of Medicine and Nursing, University of the Basque Country (UPV/EHU), 48940 Leioa, Spain; 6 Department of Urology, Basurto University Hospital, University of the Basque Country (UPV/EHU), 48013 Bilbao, Spain; jesusmiguel.undaurzaiz@osakidetza.eus (M.U.); ana.loizagairiarte@osakidetza.eus (A.L.-I.); amparo.perezfernandez@osakidetza.eus (A.P.-F.); 7 Department of Cellular Biology and Histology, Faculty of Medicine and Nursing, University of the Basque Country (UPV/EHU), 48940 Leioa, Spain; aintzane.asumendi@ehu.eus; 8 Department of Pathology, San Giovanni Bosco Hospital, 10154 Turin, Italy; claudia.manini@aslcittaditorino.it; 9 Clinical Department, Faculty of Medical Sciences, European University of Madrid, 28670 Villaviciosa de Odón, Spain; javier.angulo@universidadeuropea.es; 10 Department of Urology, University Hospital of Getafe, 28907 Getafe, Spain; 11 Department of Pathology, Cruces University Hospital, 48903 Barakaldo, Spain

**Keywords:** clear cell renal cell carcinoma, prognosis, plasma, PD-1, PD-L1

## Abstract

**Simple Summary:**

Renal cell carcinoma (RCC) is a heterogeneous and complex disease with almost no response to chemotherapy. Immune checkpoint inhibitors have achieved great clinical success but no interesting circulating markers of clinical use have developed so far in clear cell renal cell carcinoma (CCRCC). We investigate the diagnostic and prognostic role of plasma PD-1 (sPD-1) and PD-L1 (sPD-L1) proteins for the first time together with the immunohistochemical expression counterpart of these proteins within the tumor front and tumor center in the same sample of patients with renal cancer undergoing surgery. We also investigate these plasma and tissue markers in the population of metastatic patients according to International mRCC Database Consortium (IMDC) prognostic groups and the response to systemic therapy. The independent role of sPD-L1 as a predictor of prognosis and treatment response is demonstrated.

**Abstract:**

(1). *Background*: Immunohistochemical (IHC) evaluation of programmed death-1 (PD-1) and its ligand (PD-L1) is being used to evaluate advanced malignancies with potential response to immune checkpoint inhibitors. We evaluated both plasma and tissue expression of PD-1 and PD-L1 in the same cohort of patients, including non-metastatic and metastatic clear cell renal cell carcinoma (CCRCC). Concomitant plasma and tissue expression of PD-1 and PD-L1 was evaluated with emphasis on diagnostic and prognostic implications. (2) *Methods*: we analyzed PD-1 and PD-L1 IHC expression in tumor tissues and soluble forms (sPD-1 and sPD-L1) in plasma from 89 patients with CCRCC, of which 23 were metastatic and 16 received systemic therapy. The primary endpoint was evaluation of overall survival using Kaplan-Meier analysis and the Cox regression model. Plasma samples from healthy volunteers were also evaluated. (3) *Results*: Interestingly, sPD-1 and sPD-L1 levels were lower in cancer patients than in controls. sPD-1 and sPD-L1 levels and their counterpart tissue expression both at the tumor center and infiltrating front were not associated. Higher expression of both PD-1 and PD-L1 were associated with tumor grade, necrosis and tumor size. PD-1 was associated to tumor stage (pT) and PD-L1 to metastases. sPD-1 and sPD-L1 were not associated with clinico-pathological parameters, although both were higher in patients with synchronous metastases compared to metachronous ones and sPD-L1 was also higher for metastatic patients compared to non-metastatic patients. sPD-1 was also associated with the International Metastatic Renal Cell Cancer Database Consortium (IMDC) prognostic groups in metastatic CCRCC and also to the Morphology, Attenuation, Size and Structure (MASS) response criteria in metastatic patients treated with systemic therapy, mainly tyrosine-kinase inhibitors. Regarding prognosis, PD-L1 immunostaining at the tumor center with and without the tumor front was associated with worse survival, and so was sPD-L1 at a cut-off >793 ng/mL. Combination of positivity at both the tissue and plasma level increased the level of significance to predict prognosis. (4) *Conclusions*: Our findings corroborate the role of PD-L1 IHC to evaluate prognosis in CCRCC and present novel data on the usefulness of plasma sPD-L1 as a promising biomarker of survival in this neoplasia.

## 1. Introduction

Clear cell renal cell carcinoma (CCRCC) is a very prevalent disease and a clinical problem of major concern in Western countries due to its biological aggressiveness and its well-known resistance to chemotherapy and radiotherapy regimes [1,2,3]. Traditionally, radical surgery coupled with early diagnosis has been the only strategy with a direct impact on patient survival [4]. CCRCC is a model of hypoxia-related disease. *VHL* gene malfunction is detected in the overwhelming majority of the cases, resulting in a pseudo-hypoxic status that promotes angiogenesis [5]. The implementation of antiangiogenic therapies with tyrosine kinase inhibitors has improved the prognosis of many of these patients [6,7]. However, its efficacy is limited due to the development of resistant-to-therapy cell clones [8].

Immune checkpoint blockade of PD-1 and its ligand PD-L1 have been implemented in advanced lung, renal (CCRCC) and bladder carcinomas, as well as in melanoma, with promising results in several trials [9,10]. In CCRCC the immunohistochemical evaluation is selectively performed in the intratumor lymphoid inflammatory infiltrates. However, the patient selection for such a form of therapy is difficult, since this evaluation is subjected to interobserver variability [11]. In fact, up to 17% of patients with negative immunohistochemistry results do respond to this therapy [12]. Other important limitations for the development of immune checkpoints inhibitors targeting the PD-1 pathway are that responses rates are low and biomarkers are needed for the prediction of treatment responses [13,14].

To overcome the aforementioned difficulties, composite biomarkers have been investigated including tumor mutational burden, profiling of tumor infiltrating lymphocytes, molecular subtypes and the characterization of ligand PD-L2. Distinct tumor microenvironment immune types have been described, mainly based on the level of CD8A and PD-1 expression, with the intention to standardize a more comprehensive score to be used as a prognostic marker [15]. Combination with other composite biomarkers is currently under investigation [16]. Another interesting strategy to maximize the clinical benefit and predict treatment toxicity is the characterization of gastrointestinal microbiome [17]. Surprisingly, not much attention has been given to the evaluation of soluble PD-1 (sPD-1) and PD-L1 (sPD-L1) in plasma as potential biomarkers in patients with CCRCC, a heterogeneous neoplasm in serious need of identification of molecular markers that clinicians could use to facilitate an earlier diagnosis, to monitor the disease and to predict prognosis and clinical response to different therapies.

We evaluate plasma and tissue expression of PD-1 and PD-L1 in the same cohort of patients and analyze the relationship between them, also taking into account the non-metastatic and metastatic samples. Within metastatic CCRCC, plasma and tissue expression of PD-1 and PD-L1 were analyzed according to the IMDC risk classification and also according to the Morphology, Attenuation, Size and Structure (MASS) response criteria in patients receiving systemic therapy for metastatic disease. Also, we provide a very interesting simultaneous evaluation of sPD-1 and sPD-L1 and its concomitant expression in the tumor center and infiltrating front, with emphasis on the prognostic implication of these categories. The potential use of sPD-L1 as a tumor marker itself is also discussed, and its relation to other clinical and pathological variables that predict prognosis in CCRCC and treatment response in metastatic CCRCC, according to MASS criteria, is investigated.

## 2. Results

### 2.1. PD-L1 and PD-1 Tissue Expression and Plasma Levels Are Not Correlated with the Gender and Age of CCRCC Patients

To assess whether the expression in tumors and plasma levels of these biomarkers varies according to the gender or age of the patients, the non-parametric Rho Spearman test was performed. There was not any statistically significant correlation in any case (Table S1). Therefore, it can concluded that the sample has no gender or age bias.

### 2.2. The Expression of PD-L1 and PD-1 at the Tumour Centre and at the Infiltrating Front Is Correlated

**Figure 1:**
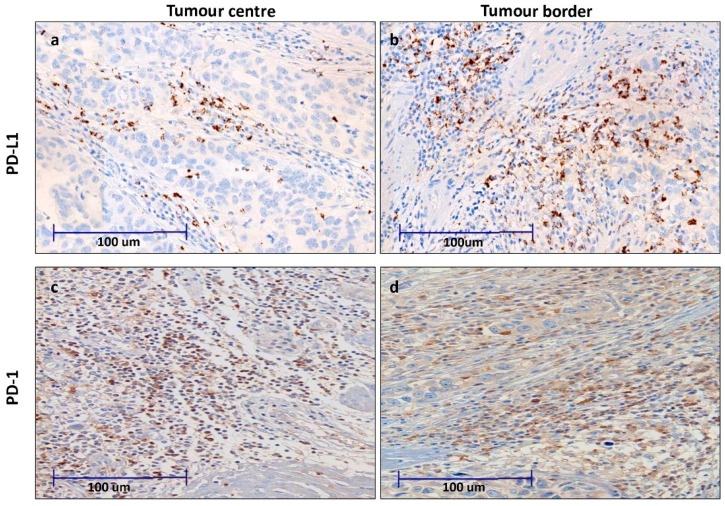
Immunohistochemical expression of PD-1 (sPD-1) and PD-L1 (sPD-L1) staining in inflammatory cells in clear cell renal cell carcinoma (CCRCC) samples, both in the tumor center (**a**,**c**) and infiltrating front (**b**,**d**).

We analyzed the expression of PD-L1 and PD-1 in lymphocytes at both the tumor center and front (Figure 1). The expression correlated positively in all cases (Table S2). Thus, the higher the percentage of PD-L1 or PD-1 positives at the tumor center, the higher the percentage was at the tumor front. Moreover, PD-L1 correlated positively with the expression of PD-1.

Although there was a significant positive correlation between the expression of both biomarkers at the tumor center and edge, this does not mean that there was a concomitant expression in all cases. Therefore, we also evaluated the simultaneous positive staining of PD-L1 and PD-1 at both areas of tumors and stratified the rest of data, taking this characteristic into account. Thus, simultaneous positivity of PD-L1 at tumor center and front was found to be correlated with simultaneous expression of PD-1 at both areas (Table S2).

### 2.3. Plasma PD-L1 Levels Are Lower in CCRCC Patients than in Control Subjects

Plasma levels of sPD-L1 and sPD-1 from CCRCC patients were compared to plasma from 46 controls (Table 1). sPD-L1 levels were significantly lower in patients than in healthy subjects. Plasma sPD-1 levels showed high variability both in patients and in controls. These levels were higher in patients than in controls; however, the result was not statistically significant.

We also aimed to analyze the association between plasma levels of these two biomarkers according to their expression at the tumor center, at the infiltration front and, simultaneously, at both areas (Table 2). We observed higher plasma PD-L1 levels in patients whose tumors were PD-L1 positive at the tumor center, border and at both areas. However, this trend was not statistically significant. We did not find any significant association between sPD-1 levels and PD-1 expression in CCRCC tissues.

### 2.4. Tissue Expression of PD-L1 and PD-1 as Well as Plasma sPD-L1 and sPD-1 Are Associated with CCRCC Aggressiveness

**Figure 2:**
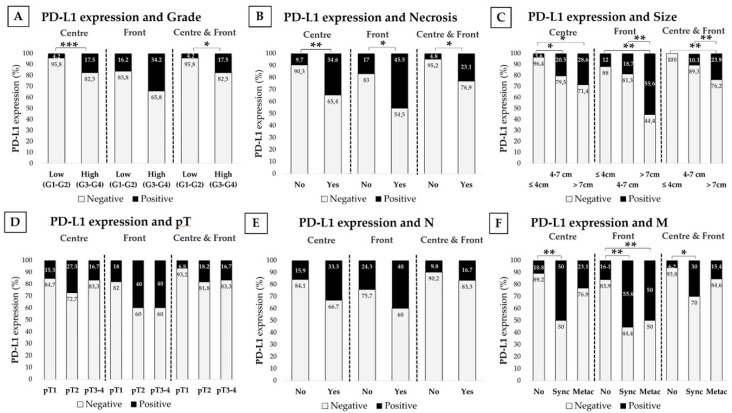
Immunohistochemical PD-L1 staining in terms of the CCRCC aggressiveness. PD-L1 immunostaining at the tumor center, infiltrating front and simultaneously at both areas depending on histological grade (**A**), tumor necrosis (**B**), diameter (**C**), local invasion or pT (**D**), lymph node metastasis or N (**E**), and distant metastasis or M (**F**). PD-L1 staining intensity was scored as negative or positive. Chi-Square test * *p* < 0.05; ** *p* < 0.01, *** *p* < 0.001.

**Figure 3:**
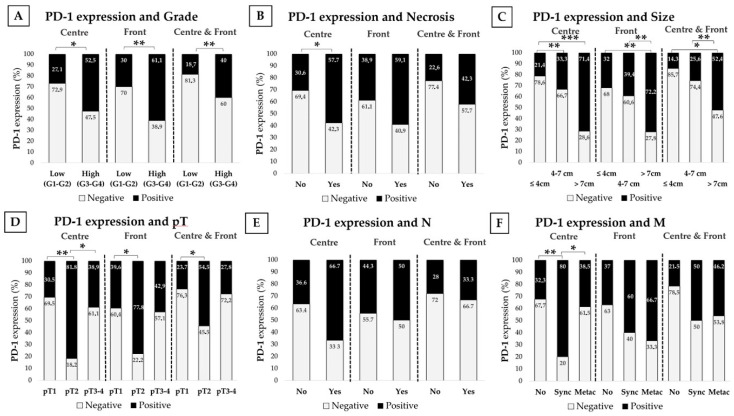
Immunohistochemical PD-1 staining according to CCRCC aggressiveness. PD-1 immunostaining at the tumor center, infiltrating simultaneously at both areas depending on the histological grade (**A**), tumor necrosis (**B**), diameter (**C**), local invasion or pT (**D**), lymph node metastasis or N (**E**) and distant metastasis or M (**F**). PD-1 staining intensity was scored as negative or positive. Chi-Square test * *p* < 0.05; ** *p* < 0.01, *** *p* < 0.001.

We stratified results by clinical parameters tightly related to tumor aggressiveness such as the Fuhrman histological grade, tumor necrosis, size, local invasion (pT), presence/absence of affected lymph nodes (N) and time of presentation of distant metastasis (M). Data are shown in Figure 2 and Figure 3. Data in metastatic patients was also evaluated according to IMDC categories predictive of prognosis and also in metastatic patients receiving systemic therapies, mainly tyrosine kinase inhibitors (TKIs) in sequential use (Table S3), results were evaluated according to the tumor response to treatment following the MASS criteria.

#### 2.4.1. PD-L1 and PD-1 Expression Is Higher in High-Grade Tumors

Tumors were stratified as having a low Fuhrman grade (G1–G2) and a high-grade (3–4). High-grade CCRCCs showed higher PD-L1 and PD-1 expression than low-grade tumors, both at the center and at the infiltrating front. Simultaneous positive expression at both areas was also higher in high grade CCRCCs (Figure 2A or Figure 3A).

Plasma sPD-L1 and sPD-1 showed opposite pattern, with higher levels in patients with low grade tumors; however, these results were not statistically significant (Table 3).

#### 2.4.2. PD-L1 and PD-1 Are Highly Expressed in CCRCC Tumors with Necrosis

These series had 26 necrotic tumors. PD-L1 expression at the center and border was higher in these tumors. Simultaneous expression of PD-L1 at both areas was more frequent in necrotic CCRCCs (Figure 2B). PD-1 expression showed a similar staining pattern, but data only reached statistical significance at the tumor center (Figure 3B). Plasma sPD-L1 and sPD-1 levels did not show any significant difference depending on the necrosis status of CCRCCs (Table 3).

#### 2.4.3. PD-L1 and PD-1 Positive Staining Is More Frequent in Larger CCRCCs

We classified tumors in three groups: tumors with 4 cm or smaller, 4 to 7 cm and larger than 7 cm. We observed that the larger the tumor was, the higher the positive staining of both biomarkers (Figure 2C or Figure 3C). However, these results in tumor tissues were not reflected in plasma, since sPD-L1 and sPD-1 did not vary significantly (Table 3).

#### 2.4.4. PD-1 Expression Is Associated to Local Invasion (pT)

The limited number of pT4 cases led us to stratify the local invasion in three groups: pT1 (organ-confined tumors smaller than 7 cm), pT2 (organ-confined tumors larger than 7 cm) and pT3-pT4 (non-organ-confined tumors). Percentages of PD-1 positive staining were significantly higher in pT2 tumors than in pT1 (Figure 3D). PD-L1 staining was also higher in pT2 tumors; however, data did not reach statistical significance (Figure 2D). Plasma analyses did not provide any significant results (Table 3).

#### 2.4.5. PD-L1 and PD-1 Tissue Expression and Plasma sPD-L1 and sPD-1 Are Higher in Patients with Synchronous Distant Metastasis

Data were also stratified by lymph node (N) and distant metastasis (M). Plasma sPD-L1 levels were higher in patients with lymph node invasion; however, the number of patients with this characteristic was limited (*n* = 6) and the result did not reach statistical significance (Table 3). PD-L1 and PD-1 expression in tissue and sPD-1 in plasma did not show any relevant difference (Figure 2E or Figure 3E).

With respect to distant metastases, we first compared primary tumors with (M1) or without (M0) metastases at the moment of the first diagnosis of CCRCC. PD-L1 expression at the tumor center (Chi-square test, *p* = 0.004), front (*p* = 0.029) and simultaneously at both areas (*p* = 0.03) was higher in primary tumors with onset as metastatic lesions than in not metastasized ones. PD-1 in the center of tumors also predicted metastasis (*p* = 0.005) (data not shown in figures or tables).

We also classified distant metastases as early synchronous (metastases that debuted within 6 months of the first primary cancer) and late metachronous (relapse of the disease with distant metastases more than 6 months later), and compared them with tumors that did not metastasize during follow-up. Thus, primary CCRCCs with synchronous metastases showed higher percentages of positive staining of PD-L1 (tumor center, front and simultaneous) and PD-1 (center) than in tumors that did not metastasize (Figure 2F or Figure 3F). PD-L1 in tumor front was also higher in metachronous ones than in tumors without metastases.

Plasma analyses showed that sPD-L1 levels were higher in patients that manifested with metastasis at the onset of the disease (M0: 857 ± 157 ng/mL vs. M1: 1014 ± 191, Mann-U test, *p* = 0.017). Furthermore, levels were also higher in patients with synchronous metastases than in patients without (Table 3). Both sPD-L1 and sPD-1 levels were also higher in patients with early metastases than with metachronous ones (Table 3).

### 2.5. PD-L1 and PD-1 Expression and Plasma Levels in Terms of the Overall Survival (OS) of CCRCC Patients

**Figure 4:**
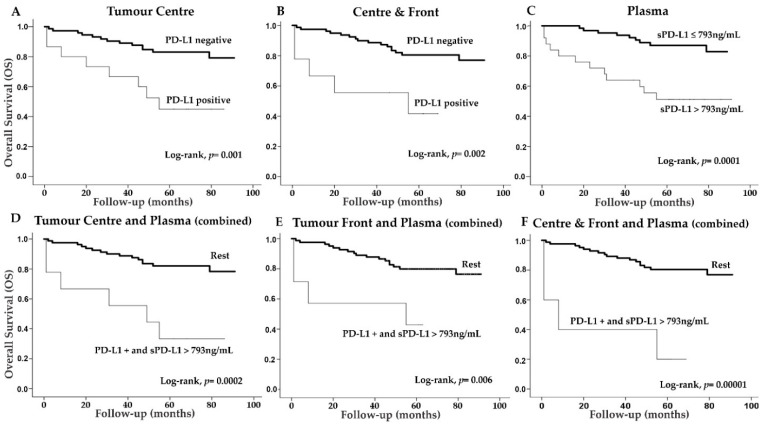
Immunohistochemical PD-L1 expression and plasma sFAP levels according to CCRCC patients’ 5-year overall survival (OS). Kaplan-Meier curves and univariate Log-rank test showed that PD-L1 expression at tumor center (**A**) and concomitant expression at center and border (**B**) is associated to worse OS. (**C**) CCRCC patients with sPD-L1 above 793 ng/mL had worse OS. The expression of PD-L1 at tumor center (**D**), front (**E**) or at both areas (**F**) together with plasma sPD-L1 levels above 793 ng/mL are associated with worse OS.

PD-L1 positive immunostaining at the tumor center and simultaneously at both the center and front was associated with a worse 5-year OS of CCRCC patients (Figure 4A,B). The expression of PD-L1 at the infiltrating front showed a similar result but it did not reach statistical significance (Log-rank test, *p* = 0.068). PD-1 expression at the center (Log-rank test, *p* = 0.29), front (*p* = 0.24) and concomitantly at both areas (*p* = 0.23) was not associated to OS.

A Classification and Regression Tree (CRT) was employed to obtain cut-off values of plasma sPD-L1 and sPD-1 for OS analyses (Figure S1). A plasma sPD-L1 value of 793 ng/mL determined two nodes with significant differences in the percentage of dead patients: 14.1% of deaths in the group of patients had plasma levels below this cut-off and 48.8% of deaths in the group had sPD-L1 levels above this cut-off (*p* = 0.047) (Figure S1A). Thus, Kaplan-Meier curves demonstrated that CCRCC patients with sPD-L1 levels above 793 ng/mL had worse 5-year OS than patients with lower levels (Figure 4C).

With regard to sPD-1, the CRT selected a cut-off value of 27ng/mL (*p* = 0.017) (Figure S1B). Kaplan-Meier curves showed a trend towards worse survival in CCRCC patients with plasma sPD-1 levels below this cut-off; however, the difference did not reach statistical significance (Log-rank test, *p* = 0.073).

Tumors and plasmas were obtained from the same patients. Therefore, taking into account the significant results with PD-L1 and its soluble isoform predicting patients’ 5-year OS, we also performed Kaplan-Meier curves by combining data of tissue expression and plasma levels. Thus, two groups were created: (1) PD-L1 positive cases at the center of tumors, at the infiltrating front or simultaneously at both areas, together with sPD-L1 levels above 793 ng/mL; and (2) the rest of the possible combinations (PD-L1-/sPD-L1 ≤793 ng/mL; PD-L1-/sPD-L1 >793 ng/mL; PD-L1+/sPD-L1 ≤793 ng/mL). CCRCC patients with tumor PD-L1 positivity and plasma levels above 793 ng/mL had significantly worse 5-year OS than patients with the rest of combinations (Figure 4D–F).

Multivariate Cox regression analyses were performed to determine whether PD-L1 expression in tumor center, concomitantly at both center and front, sPD-L1 plasma levels (cut-off 793 ng/mL) or the combination of both isoforms are independent prognostic factors for 5-year OS. The logistic model resulting from a backward Wald stepwise elimination of variables revealed that the expression of PD-L1 at the tumor center, concomitant expression at both areas and plasma sPD-L1 were independent prognostic factors for 5-year OS (Table 4). Moreover, combinations of PD-L1 positivity in tumor tissues and plasma sPD-L1 were also explanatory independent variables for patients’ OS. Complete multiple Cox regression is shown as supplementary material (Table S4).

### 2.6. PD-L1 and PD-1 Tissue Expression and Plasma Levels in Patients with Metastatic CCRCC According to IMDC Model and Response to Therapy

Twenty-three patients with metastatic CCRCC were stratified according to the IMDC model for classification of patients at different risks of death. PD-L1 and PD-1 tissue expression and plasma levels of sPD-L1 and sPD-1 were also stratified according to IMDC categories. With the limited number of patients in this subseries, the percentage of patients with positive PD-L1 and PD-1 tissular expression did not associate with IMDC groups; however, if the favorable and intermediate groups are pooled together, then PD-L1 expression in tumor center was higher in patients with poor prognosis and approached statistical significance (*p* = 0.056) (Table 5). Also, median sPD-L1 levels almost correlated with IMDC groups (*p* = 0.062) and did so again when favorable and intermediate median sPD-L1 levels discriminated against prognostic groups (*p* = 0.021) (Table 5).

Sixteen patients received systemic therapy for metastatic CCRCC and response to therapy was evaluated according to the MASS criteria. PD-L1 and PD-1 expression and sPD-L1 and sPD-1 levels were investigated according to the three categories of favorable, indeterminate and unfavorable responses (Table 6).

The percentage of patients with positive PD-L1 and PD-1 tissular expression did not associate with MASS response groups to systemic therapy; however, if the indeterminate and unfavorable response groups are pooled together, then PD-L1 expression in the tumor front was more often negative and the association approached statistical significance (*p* = 0.079) (Table 6). However, median sPD-L1 levels correlated with the different IMDC groups (*p* = 0.014) and did so again when favorable and intermediate are gathered (*p* = 0.021). The discrimination level was even enhanced if indeterminate and unfavorable responses were pooled together and compared to patients with a favorable response (*p* = 0.005). These data suggest that sPD-L1 could be a marker of treatment response in patients with metastatic CCRCC receiving systemic therapy (Table 6).

## 3. Discussion

The T-cell coinhibitory receptor programmed death (PD-1) protein and one of its ligands, PD-L1, play an important role in the evasion of the immune system by tumor cells. Both PD-1 and PD-L1 suppress T cell function and immune tolerance [18]. Recent clinical commercialization of PD-1 pathway inhibitors (nivolumab, pembrolizumab, atezolizumab, durvalumab, avelumab) has raised interest in PD-1 and PD-L1 expression as potential markers of response to immune checkpoint therapy in several malignancies, including CCRCC [19]. Identification and validation of biomarkers will be crucial to optimize first-line selection of treatment and also treatment sequences.

In this sense, it has been demonstrated that PD-1 and PD-L1 expression is associated with adverse clinico-pathological features in CCRCC, such as a large tumor size, high nuclear grade, tumor necrosis and presence of sarcomatoid differentiation [20]. What is more, PD-1 expression has been suggested as one of the most interesting biomarkers denoting poor outcomes in patients with metastatic CCRCC receiving molecular targeted therapies, while conflicting results have been shown for PD-L1 in the same population [20,21]. Both PD-1 and PD-L1 are expressed in intra-tumor inflammatory lymphocytes [22]. PD-1 and PD-L1 expression associates with CD4+, CD8+ and FOXP3+ tumor infiltrating lymphocytes related to poor survival in CCRCC [20,23,24]. However, the identification of patients that are likely to obtain a benefit from PD-1/PD-L1 inhibition therapy remains a challenge [25].

Metastatic CCRCC with a long-term response to sunitinib has been characterized as a distinct phenotype independently associated with low PD-L1 expression [26]. However, the inherent heterogeneity of CCRCC includes a very variable expression of positive and negative regions of PD-L1 expression within each tumor [27]. Also, differential expression of PD-1 and PD-L1 has been confirmed between primary and metastatic sites within the same case [28,29,30]. This conflicting scenario can be worsened as the different expression across primary and metastatic tumor for PD-L1 could be associated with metastatic tumor timing. In fact, larger differences between their primary and metastatic tumor pairs have been detected in synchronous metastatic patients in comparison to the metachronous metastatic ones, and this could be explained by the fact that distant metachronous metastasis may have evolved independently of the primary tumor [31].

PD-L1 expression has been used as surrogate marker of response to immune checkpoint inhibitors and, indirectly, as marker of prognosis as well. In fact, despite all the limitations mentioned to evaluate responses to therapy based on PD-1/PD-L1 expression, a tendency towards a higher PD-L1 expression has been confirmed in responders but without a good correlation [32]. For this reason, PD-L1 assessment is not required so far to initiate immune checkpoint inhibition therapy in patients with CCRCC. On the other hand, strong evidence is accumulating to consider PD-L1 expression as a likely strong prognosticator in patients with CCRCC not only in metastatic cases receiving anti-PD-1 antibodies, but also receiving sunitinib or pazopanib [33]. In the series that we present here, PDL-1 expression in CCRCC and sPD-L1 levels were predictors of overall survival, and the combination of both tissue expression and plasma levels was an independent predictor of prognosis. It should be stressed that most of the patients in this series were only treated surgically and therefore, we cannot directly infer that PD-L1 (either tissular or plasmatic) is an independent prognostic marker in patients with metastatic CCRCC treated with systemic therapies. Also, as the tissue and plasma samples analyzed in this series belong to the TKI era (checkpoint immune inhibitors were only used in a small number of patients after progression on TKI). Even though the number of patients with metastatic CCRCC in this series is small, we can confirm that PD-L1 expression in tumor center is higher in metastatic patients within the IMDC poor prognosis group (*p* = 0.056) and also that sPD-L1 levels better discriminate poor prognosis for this population of (*p* = 0.021).

Circulating sPD-L1 can be determined by ELISA in normal human serum and in supernatants of different cells including CD4+, CD8+, CD19+, CD14+ and CD56+ T cells, and may play an important role in immunoregulation [34]. sPD-L1 have been described in several malignancies including renal cell cancer, pancreatic cancer, rectal cancer, B-cell lymphoma, multiple myeloma and melanoma [35,36,37,38,39]. It has been hypothesized that sPD-L1 may act as a paracrine negative immune regulator within the tumor [40]. However, the sources of sPD-L1 in patients with cancer is unclear, as it may derive from protumor inflammatory responses, antitumor immune-responses and also intrinsic splicing activities in tumor cells. It is also unclear whether sPD-L1 is associated with clinical characteristics such as patient age, sex or treatment response. In our series sPD-L1 is higher in controls than in patients with CCRCC and the level of sPD-L1 in cancer patients is associated with metastatic disease, but not with conventional prognosticators of CCRCC. Interestingly, higher levels of sPD-L1 in CCRCC are an independent predictor of prognosis. Other authors have investigated the role of several immune checkpoint-related proteins as predictors of tumor recurrence and survival in CCRCC and sustain sTIM3 and sBTLA, but did not predict worse survival for sPD-L1 [41].

According to our experience, both PD-1 and PD-L1 immunohistochemical expression are associated with well-recognized histopathologic parameters of tumor aggressiveness and PD-L1 is also an independent marker of prognosis in our series, both on the tumor center and invasive fronts. Notably, this is a population of patients with CCRCC including all stages and not necessarily treated with antiangiogenic therapy or immune checkpoint inhibition therapy. What is more, we have simultaneously evaluated PD-1 and PD-L1 both in the tumor and serum of the same cohort of patients and have confirmed that sPD-L1 is definitely an independent prognostic factor that is non-associated with the tumor size, Fuhrman grade or histopathological staging. Multivariate analysis revealed that sPD-L1 > 793 ng/mL is associated with worse survival (HR 8.67), together with pT category (HR 2.24) or presence of metastasis (HR 2.83). We also confirmed a major variation in sPD-L1 levels according to the time of the metastatic event, with a much higher expression in synchronous metastases than in metachronous ones. No less interesting is the fact that a positive PD-L1 expression in the tumor center and the invading tumor front— as well as as PD-L1 level > 793 ng/mL—leads to a worse overall survival rate in CCRCC patients. However, what is even more interesting in our experience is that sPDL-1 levels appear to represent a good surrogate of a response criteria to systemic therapy administered in metastatic CCRCC. In this context, there are many limitations to consider when deciding to treat CCRCC patients with immune checkpoint inhibitors based on the immunohistochemical detection of PD-1/PD-L1 positivity alone [11,27], while the search of markers to anticipate the response to immunotherapy continues [42,43,44]. A new trial (UMIN000027873) has been recently launched to evaluate the therapeutic effect of nivolumamb as a second-line therapy for advanced CCRCC based on the concentrations of serum sPD-L1. The hypothesis of this study is that patients with high blood levels of sPD-L1 will experience a greater therapeutic effect during nivolumab treatment [45].

The limitations of our study include its retrospective nature, despite the fact that the cases were prospectively followed after tumor and serum samples were obtained. Patients were treated using state-of-the art procedures, and size of the sample was also relatively small, especially when different subsets of patients were specifically analyzed. Also, our finding that sPD-1 levels in controls are higher than in CCRCC patients could be explained by a confounding effect of disparity levels in CCRCC patients due to the fact that the sample includes patients with all stages of disease. It also could have been due to inappropriateness of the control sample as a result of unknown factors. Regardless, we did not test the hypothesis that sPD-L1 can be a tumor marker for the diagnosis of CCRCC, but we did show evidence supporting the idea that it can be a good marker to evaluate prognosis for CCRCC patients when they are taken as a whole, and also in the subset of metastatic patients being treated with the IMDC model. Also, we support its use as a marker of prognosis in metastatic patients treated with systemic therapies, mainly TKIs.

Future studies should try to evaluate the role of sPD-L1 and other soluble immune checkpoint-related proteins to elucidate their role as intrinsic tumor markers with utility in prognostic evaluation involving CCRCC as a malignancy without markers of clinical value, despite the great therapeutic success that has been achieved in the last decade.

## 4. Materials and Methods

The present study including all of its experiments comply with current Spanish and European Union legal regulations. The Basque Biobank for Research-OEHUN (www.biobancovasco.org) was employed the source of samples and data from patients that could be used for research purposes. Each patient signed a specific document which had been approved by the Ethical and Scientific Committees of the Basque Country Public Health System (Osakidetza) (PI + CES-BIOEF 2018-04).

### 4.1. Patients

Plasma samples and tumor tissues were obtained from 89 CCRCC patients that were surgically treated at Basurto University Hospital from 2012 to 2016. The plasma samples were preoperatively collected for the study. Patients with non-metastatic CCRCC were treated surgically and patients with metastatic disease received nephrectomy and systemic therapy according to their ICDM classification, age and clinical condition.

Sixty patients were males (mean age: 60.83 years; range: 36–82) and 29 were females (mean age: 62.69; range: 32–80). Pathological characteristics are summarized in Table 3. Plasma from 46 healthy volunteers with no clinical history of neoplastic diseases was used as control samples (male/female 28/18, age 55.8/61.8 years).

Samples from the center (*n* = 88) and the infiltration front (*n* = 75) of tumors from these patients were distinguished in the histopathological department and included in tissue microarrays (TMAs) for further immunohistochemical analyses. American Joint Committee on Cancer (AJCC) [19] and Furhman’s [20] methods were applied to assign the relevant stage and grade, respectively.

During the follow-up (mean: 59.9 months, range: 1–91 months), 21 patients were found to no longer be alive and 68 were still alive. All patients were prospectively followed until death or the last-follow-up. The cause and date of death was taken as specified in clinical records and overall survival (OS) was investigated.

### 4.2. IMDC Model and MASS Response Criteria for Patients with Metastatic CCRCC

The International mRCC Database Consortium (IMDC) represents the largest collection of real-world data on patients with advanced kidney cancer treated with targeted therapies. The IMDC prognostic model has been used to stratify patients in contemporary clinical trials and to provide risk-directed treatment selection in everyday clinical practice. This model classifies metastatic patients into three categories at different risk of death: favorable, intermediate and poor risk [46]. We used the IMDC to evaluate the group of patients with metastatic CCRCC in this series (*n* = 23).

In order to evaluate the response assessment to systemic therapy in metastatic CCRCC receiving treatments other than nephrectomy (*n* = 16), the Morphology, Attenuation, Size and Structure (MASS) criteria was used to distinguish between the three categories of patients. Patients with a favorable response to therapy are those with no new lesions displayed on imaging modalities and any of the following outcomes: i. A decrease in the tumor size of ≥20%; ii. One or more predominantly solid enhancing lesions showed marked central necrosis or marked decreased attenuation (≥40 Hounsfield units). Patients with an unfavorable response are those with either: i. An increase in the tumor size of ≥20% in the absence of marked central necrosis or marked decreased attenuation; ii. New metastases, marked central fill-in or new enhancement of a previously homogeneously hypoattenuating non-enhancing mass. Patients with an indeterminate response are those who do not fit the criteria for favorable or unfavorable responses [47].

### 4.3. Immunohistochemistry

PD-L1 and PD-1 was analyzed in formalin-fixed and paraffin-embedded material using specific antibodies (PD-1 (Ventana, clone NAT105, ready-to-use) and PD-L1 (Ventana, clone SP-142, ready-to-use)). Immunostaining was performed using an automated immunostainer (Benchmark Ultra, Ventana, Roche, AZ, USA) following the protocols recommended by the manufacturer.

We documented the presence (+) or absence (−) of PD-L1 and PD-1 immunolabels in inflammatory cells [27] using a Nikon Eclipse 80i microscope (Nikon, Tokyo, Japan). All specimens were independently evaluated by two observers; in the event of discrepancies, samples were re-evaluated to arrive at a final conclusion.

### 4.4. ELISA Assays

Levels of soluble PD-L1 and PD-1 were evaluated using the human B7-H1 and PD-1 DuoSet ELISA kits (R&D Systems, DY156 and DY1086, respectively) according to the manufacturer’s protocols [37]. Briefly, 96 well plates were coated with capture antibodies diluted in PBS and incubated overnight at 4 °C. After washing, plates were blocked in order to avoid unspecific binding. Standards (100 µL) together with optimized plasma sample dilutions (1/8 for sPD-L1 and 1/4 for sPD-1) and controls were added to the wells and incubated for 2 h at room temperature (RT). After washing the plate, 100 µL/well of biotinylated detection antibody was added and incubated for 1 h at RT. Subsequently, Streptavidin-HRP A solution was added and the mixture was incubated for 20 min. Finally, following multiple washes, the wells were incubated with 100 µL/well of Substrate Solution and were stopped after 20 min with 2N H2SO4. The readout was made by reading the absorbance at 450 nm with a FluoStar Optima plate reader (BMG Labtech). The amount of protein of interest in the sample was estimated using a standard curve after applying the dilution factor.

### 4.5. Statistical Analysis

The statistical analysis was performed by using SPSS^®^ 24.0 software. In order to assess whether data obtained from the tissue and plasma samples followed a normal distribution, we applied a Kolmogorov-Smirnov test. Based on this information, data were further analyzed using parametric or non-parametric tests.

The Spearman Rho test was used to test the correlation between tumor tissue PD-L1 and PD-1 expression, sPD-L1 and sPD-1 levels and patient age and gender. Comparison of plasma levels of sPD-L1 and sPD-1 between two groups or more (respectively) was carried out using the Mann-Whitney (Mann-U) and Kruskal-Wallis tests. To analyze categorical tissue expression of PD-L1 and PD-1 (negative/positive) and to test the association of differences with pathological variables, we used the Chi-square (χ2) test.

Overall survival (OS) analyses were performed following the establishing of groups by cut-off points, following different methods: (I) for tissue analyses, cut-off points were based on the categorical expression of PD-L1 and PD-1 (negative (<1% staining) vs. positive (≥1% staining)); (II) a classification and regression tree (CRT) method was employed for the analysis of plasma sPD-L1 and sPD-1; (III) in order to evaluate the OS of CCRCC patients, Kaplan-Meier curves and log-rank tests were utilized; (IV) to evaluate the independent effects of PD-L1 and PD-1 expression and plasma levels of soluble isoforms and pathological variables on OS, we employed multivariate analyses (the Cox regression model with the backward Wald stepwise method).

## 5. Conclusions

There is a major need to identify new molecular markers in CCRCC which are useful from the clinical perspective. We corroborated the value of PD-L1 immunostaining in lymphocytic infiltrate both in the tumor center and in the border of neoplastic tissue to predict worse overall survival in patients with CCRCC undergoing surgery which were not necessarily treated with immune-checkpoint inhibitors. We also advocate for the clinical utility of sPD-L1 level > 793ng/mL as an independent and novel predictor of prognosis in clinical practice for the same patients. In addition, we determined that the sPD-L1 level increased for IMDC prognostic groups in the population of patients with metastatic CCRCC, and was also associated with the clinical response of patients with metastatic CCRCC receiving systemic therapy.

These findings could be of primary importance because they indicate that the determination of sPD-L1 can be widely performed in clinical practice. Our results should be validated in prospective studies and possibly incorporated into predictive nomograms that have clinical transcendence in patients with CCRCC.

## Figures and Tables

**Table 1:** sPD-L1 and sPD-1 levels in plasma samples from clear cell renal cell carcinoma (CCRCC) patients and healthy controls. Values are means ± standard errors. Significant *p* value in bold.

sPD-L1 (ng/mL)			sPD-1 (ng/mL)		
CCRCC	Controls	Mann-U (*p*=)	CCRCC	Controls	Mann-U (*p*=)
902.8 ± 139.7	989.1 ± 155.9	**0.048**	1304.7 ± 306.3	941.3 ± 300.3	0.33

**Table 2:** Plasma sPD-L1 and sPD-1 levels in CCRCC patients in terms of PD-L1 and PD-1 expression in CCRCC tissues.

	**PD-L1 Expression at Tumour Centre**			**PD-L1 Expression at Infiltrating Front**		
	**Negative**	**Positive**	**Mann-U, *p*=**	**Negative**	**Positive**	**Mann-U, *p*=**
Plasma sPD-L1 (ng/mL)	849.1 ± 148.3	1182.1 ± 412.7	0.13	905.9 ± 184.5	1035.7 ± 353.1	0.99
	**PD-1 at Tumour Centre**			**PD-1 at Infiltrating Front**		
	**Negative**	**Positive**	**Mann-U, *p*=**	**Negative**	**Positive**	**Mann-U, *p*=**
Plasma sPD-1 (ng/mL)	1151.6 ± 344.4	1545.5 ± 576.5	0.61	1480.8 ± 446.6	983 ± 424.5	0.88
	**PD-L1 Expression in Both Areas**			**PD-1 Expression in Both Areas**		
	**Negative**	**Positive**	**Mann-U, *p*=**	**Negative**	**Positive**	**Mann-U, *p*=**
Plasma sPD-L1 (ng/mL)	845.1 ± 137.3	1439.5 ± 684.5	0.44	-	-	-
Plasma sPD-1 (ng/mL)	-	-	-	1383.2 ± 367.4	1103 ± 562.6	0.94

**Table 3:** Plasma sPD-L1 and sPD-1 levels in terms of pathological parameters of CCRCC aggressiveness. The Mann-Whitney test was used for comparisons between two groups and Kruskal-Wallis for more than two groups. Values are represented as means ± standard errors. ^a^ sPD-L1 synchronous vs. No, Mann-U *p* = 0.038; ^b^ sPD-L1 synchronous vs. Metachronous, Mann-U *p* = 0.008; ^c^ sPD-1 synchronous vs. Metachronous, Mann-U *p* = 0.037. Statistically significant values are highlighted in bold.

CCRCC Patients	*n*=	sPD-L1 (ng/mL)	*p*=	sPD-1 (ng/mL)	*p*=
Fuhrman Grade					
Low-Grade (G1-G2)	49	982 ± 215	0.53	1795 ± 474	0.23
High-Grade (G3-G4)	40	806 ± 168		678 ± 348	
Necrosis					
No	63	754 ± 248	0.55	1472 ± 371	0.15
Yes	26	964 ± 169		876 ± 537	
Size					
≤4 cm	28	1143 ± 353	0.37	1880 ± 644	0.95
>4 to 7 cm	39	685 ± 104		1021 ± 394	
>7 cm	22	982 ± 289		1024 ± 587	
Local Invasion (pT)					
pT1	59	896 ± 179	0.41	1467 ± 402	0.95
pT2	12	1049 ± 512		1414 ± 1089	
pT3–pT4	18	826 ± 157		760 ± 364	
Lymph node invasion (N)					
No	83	885 ± 148	0.08	1322 ± 328	0.14
Yes	6	1148 ± 305		1089 ± 524	
Distant metastasis (M)					
No	66	977 ± 184		1583 ± 395	0.14 ^c^
Synchronous	10	1014 ± 191	**0.034 ^a,b^**	824 ± 369	
Metachronous	13	438 ± 76		130 ± 68	

**Table 4:** Cox Regression model for 5-year overall survival (OS) prediction in CCRCC patients, final step of the Wald Method. Selected pathologic variables for analyses were: Fuhrman grade or G (low vs. high grade), tumor necrosis (no/yes), local invasion pT (pT1 vs. pT2 vs. pT3-pT4), lymph node metastasis N (no/yes) and distant metastases M (no vs. synchronous vs. metachronous). Exponentiation of the B coefficient (ExpB) with confidence interval (CI) is also included. Statistically significant values are highlighted in bold. PD-L1c: combination of tissue and soluble isoforms of PD-L1.

		**Tumour Centre**				**Centre-Front**				**Plasma**			
**5-Year OS**	**Variables**	***p***	**ExpB**	**CI**		***p***	**ExpB**	**CI**		***p***	**ExpB**	**CI**	
	pT	**0.04**	1.9	1	3.5	**0.09**	1.65	0.92	9	**0.004**	2.24	1.3	3.86
	N	**0.02**	4.09	1.2	13.9	**0.001**	6.68	2.1	21.4	-			
	M	**0.01**	2	1.19	3.38	**0.005**	2.07	1.24	3.46	**8 × 10^−6^**	2.83	1.68	4.75
	PD-L1	**0.06**	2.74	0.96	7.78	**0.026**	3.34	1.15	9.66	**1 × 10^−5^**	8.67	3.26	23.1
	**Tumour Centre and Plasma**					**Front and Plasma**				**Centre-Front and Plasma**			
**5-Year OS**	**Variables**	***p***	**ExpB**	**CI**		***p***	**ExpB**	**CI**		***p***	**ExpB**	**CI**	
	pT	**0.002**	4.66	1.77	12.22	**0.01**	1.83	1.34	9.12	**0.017**	3.3	1.24	8.66
	N	**0.02**	4.29	1.21	15.17	**0.002**	3.28	1.96	21.7	**0.005**	5.85	1.69	20.2
	M	**0.001**	2.33	1.4	3.89	**0.0001**	5.13	1.6	4.5	**0.0001**	2.63	1.57	4.4
	PD-L1c	**0.03**	3.5	1.09	11.28	**0.009**	2.56	1.53	19.7	**0.003**	7.98	2.05	31

**Table 5:** PD-L1 and PD-1 expression and sPD-L1 and sPD-1 levels in the subgroup of metastatic CCRCC classified according to the International Metastatic Renal Cell Cancer Database Consortium (IMDC) score (* Favorable and Intermediate pooled together). Chi-x^2^, Mann-Whitney and Kruskal Wallis tests were used. Statistically significant values are highlighted in bold.

		**Tumor Centre**			**Tumor Front**			**Plasma**
	**PD-L1 *n* (%)**				**PD-L1 *n* (%)**			**sPD-L1 (ng/mL)**
	**Variables**	**Negative**	**Positive**	**Total**	**Negative**	**Positive**	**Total**	
IMDC score	Favorable	7 (77.8)	2 (22.2)	9	3 (42.9)	4 (52.1)	7	488 ± 112.9
	Intermediate	6 (75)	2 (25)	8	5 (71.4)	2 (28.6)	7	705.4 ± 259.4
	Poor	2 (33.3)	4 (66.7)	6	1 (20)	4 (80)	5	967 ± 135.4
	Total	15	8	23	9	10	19	688.6 ± 109.5
	*p* = 0.161/*p* = 0.056 *				*p* = 0.203			*p* = 0.062/*p* = 0.021 *
		**Tumor Center**			**Tumor Front**			**Plasma**
	**PD-1 *n* (%)**				**PD-1 *n* (%)**			**sPD-1 (ng/mL)**
	**Variables**	**Negative**	**Positive**	**Total**	**Negative**	**Positive**	**Total**	
IMDC score	Favorable	5 (55.6)	4 (44.4)	9	3 (33.3)	6 (66.7)	9	154.3 ± 93
	Intermediate	3 (37.5)	5 (62.5)	8	2 (28.6)	5 (71.4)	7	618.1 ± 294.8
	Poor	2 (33.3)	4 (66.7)	6	3 (50)	3 (50)	6	650 ± 532.3
	Total	15	8	23	8	14	22	442.1 ± 182.8
	*p* = 0.637				*p* = 0.704			*p* = 0.341

**Table 6:** PD-L1 and PD-1 expression and sPD-L1 and sPD-1 levels in patients with treated metastatic CCRCC according to Morphology, Attenuation, Size and Structure (MASS) classification of response to therapy (* Favorable and Indeterminate responses pooled together; ** Indeterminate and Unfavorable responses pooled together). Chi-x^2^, Mann-Whitney and Kruskal Wallis tests were used. Statistically significant values are highlighted in bold.

		**Tumour Centre**			**Tumour Front**			**Plasma**
	**PD-L1 *n* (%)**				**PD-L1 *n* (%)**			**sPD-L1 (ng/mL)**
	**Variables**	**Negative**	**Positive**	**Total**	**Negative**	**Positive**	**Total**	
MASS Response	Favorable	6 (60)	4 (40)	10	2 (28.6)	5 (71.4)	7	387.5 ± 89.1
	Indeterminate	2 (66.7)	1 (33.3)	3	2 (66.7)	1 (33.3)	3	811.4 ± 78.1
	Unfavorable	2 (66.7)	1 (33.3)	3	2 (100)	0 (0)	2	1621 ± 442.9
	Total	10	6	16	6	6	12	698.3 ± 151.2
	*p* = 0.965				*p* = 0.164/*p* = 0.079 **			*p* = 0.014/*p* = 0.021 */*p* = 0.005 **
		**Tumour Centre**			**Tumour Front**			**Plasma**
	**PD-1 *n* (%)**				**PD-1 *n* (%)**			**sPD-1 (ng/mL)**
	**Variables**	**Negative**	**Positive**	**Total**	**Negative**	**Positive**	**Total**	
MASS Response	Favorable	5 (50)	5 (50)	10	3 (33.3)	6 (66.7)	9	268.9 ± 129.9
	Indeterminate	2 (66.7)	1 (33.3)	3	1 (33.3)	2 (66.7)	3	433.1 ± 265.7
	Unfavorable	1 (33.3)	3 (66.7)	3	3 (100)	0 (0)	3	1743.6 ± 935.1
	Total	8	8	16	7	8	15	647.1 ± 267
	*p* = 0.717				*p* = 0.117			*p* = 0.33

## Data Availability

Full data will be available from the Corresponding Author upon reasonable request.

## Supplementary Materials

The following materials are available online at https://www.mdpi.com/2072-6694/13/4/667/s1, Figure S1: Classification and Regression Tree (CRT) for both sPD-L1 and sP-D1, and CCRCC patients’ 5-year overall survival (OS), Table S1: Correlation between age and sex, and PD-L1 and PD-1 CCRCC expression and plasma levels (Spearman Rho test). PD-L1 and PD-1 expression in the centre of the tumour (c), infiltration front (f) and in both areas (cf), and plasma levels of sPD-L1 and sPD-1 were not correlated with sex and age of CCRCC patients, Table S2: Correlation between PD-L1 and PD-1 expression at the tumor center and at the infiltration front (Spearman Rho test), Table S3: Systemic therapies received by 16 patients with metastatic CCRCC, Table S4: Cox Regression model for 5-year overall survival (OS) prediction in CCRCC patients (complete table with the first step and the final step of Wald method).
