# Supplementary material for: Soluble PD-L1 Is an Independent Prognostic Factor in Clear Cell Renal Cell Carcinoma
Source: Cancers (Basel). 2021 Feb 7;13(4):667. doi: 10.3390/cancers13040667 (PMC7915750; doi:10.3390/cancers13040667)
Supplement: Supplementary file 1 [file cancers-13-00667-s001.zip › cancers-1035592-supp-XML/Suppl Figure 1 (CRT).pptx]

## Slide 1
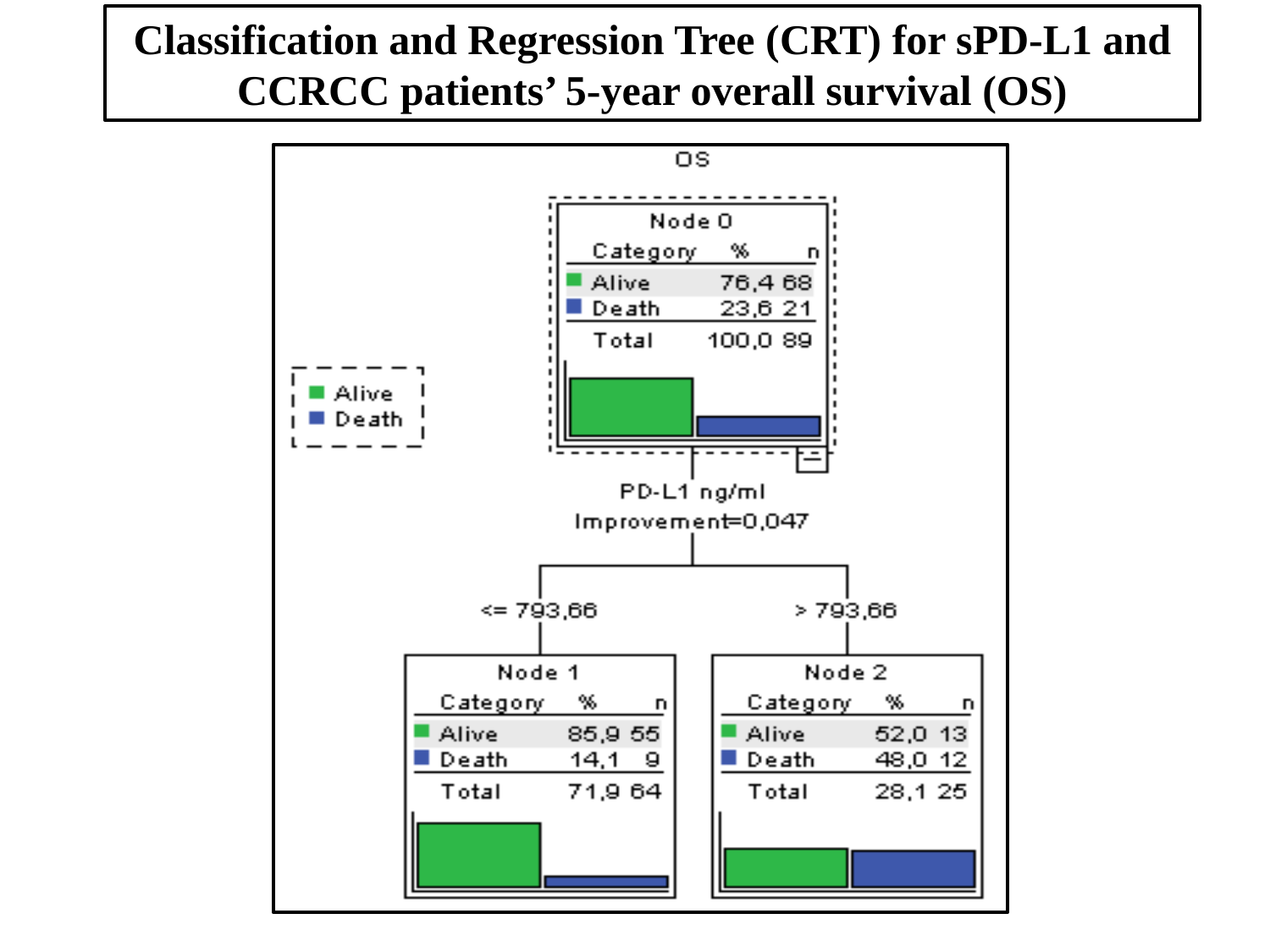

Classification and Regression Tree (CRT) for sPD-L1 and CCRCC patients’ 5-year overall survival (OS)

## Slide 2
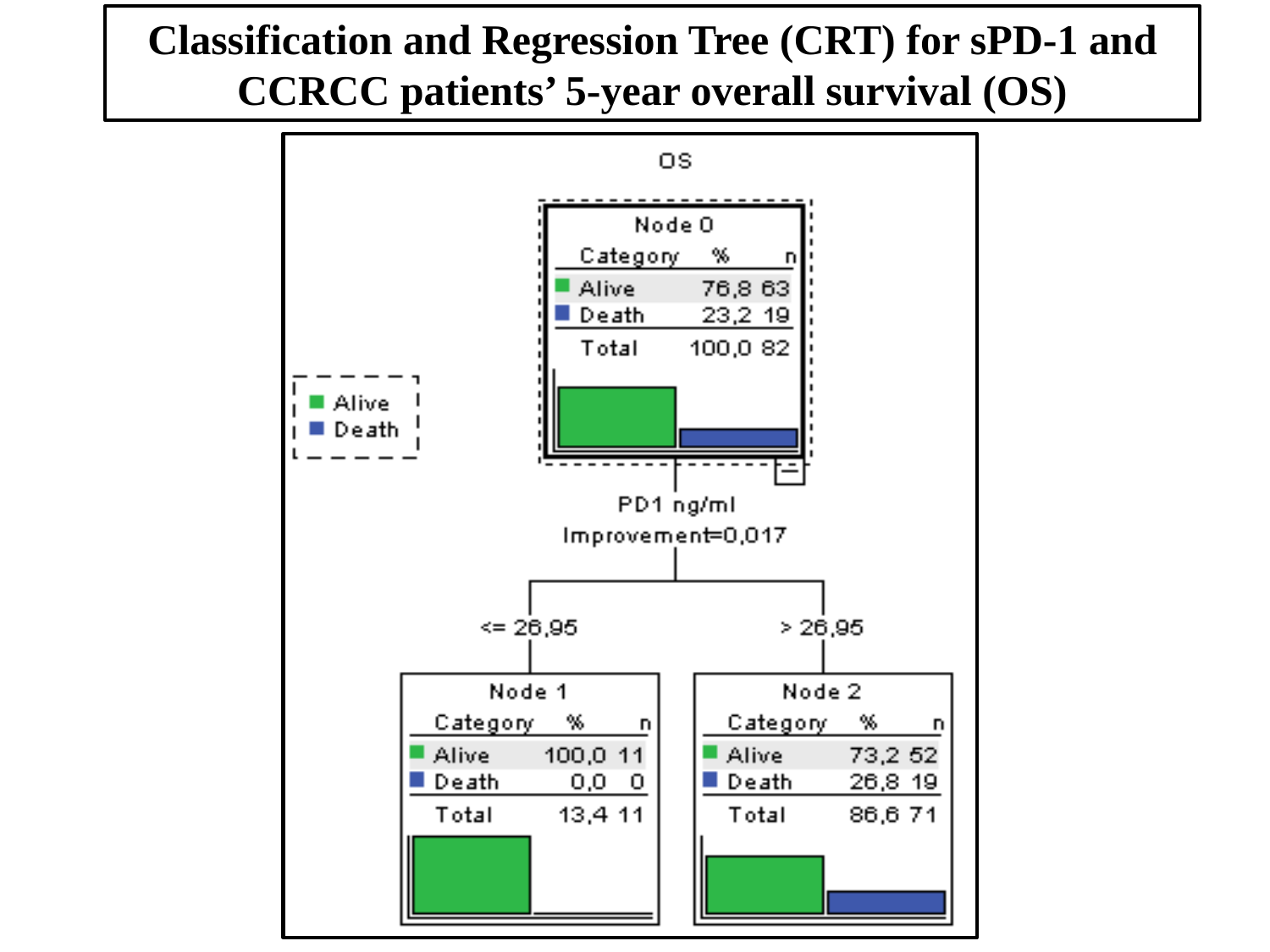

Classification and Regression Tree (CRT) for sPD-1 and CCRCC patients’ 5-year overall survival (OS)
