# Supplementary material for: Soluble PD-L1 Is an Independent Prognostic Factor in Clear Cell Renal Cell Carcinoma
Source: Cancers (Basel). 2021 Feb 7;13(4):667. doi: 10.3390/cancers13040667 (PMC7915750; doi:10.3390/cancers13040667)
Supplement: Supplementary file 1 [file cancers-13-00667-s001.zip › cancers-1035592-supp-XML/suppl Tables 1-4.docx]

Supplementary Materials: Soluble PD-L1 Is an Independent Prognostic Factor in Clear Cell Renal Cell Carcinoma

Gorka Larrinaga, Jon Danel Solano-Iturri, Peio Errarte, Miguel Unda, Ana Loizaga-Iriarte, Amparo Pérez-Fernández, Enrique Echevarría, Aintzane Asumendi, Claudia Manini, Javier C. Angulo and José I. López

**Table S1.** Correlation between age and sex, and PD-L1 and PD-1 CCRCC expression and plasma levels (Spearman Rho test). PD-L1 and PD-1 expression in the centre of the tumour (c), infiltration front (f) and in both areas (cf), and plasma levels of sPD-L1 and sPD-1 were not correlated with sex and age of CCRCC patients.

|  |  | **Tumour Tissue** | | | **Plasma** |
| --- | --- | --- | --- | --- | --- |
|  |  | **PD-L1c** | **PD-L1f** | **PD-L1cf** | **sPD-L1** |
| Sex | r = | −0.05 | 0.043 | 0.11 | −0.081 |
|  | *p* = | 0.64 | 0.71 | 0.92 | 0.7 |
| Age | r = | 0.08 | 0.019 | 0.13 | −0.022 |
|  | *p* = | 0.46 | 0.87 | 0.21 | 0.84 |
|  |  | **PD-1c** | **PD-1f** | **PD-1cf** | **sPD-1** |
| Sex | r = | −0.041 | 0.159 | 0.057 | 0.032 |
|  | *p* = | 0.7 | 0.17 | 0.6 | 0.78 |
| Age | r = | 0.018 | 0.041 | −0.008 | −0.103 |
|  | *p* = | 0.86 | 0.72 | 0.94 | 0.36 |

**Table S2.** Correlation between PD-L1 and PD-1 expression at the tumour centre and at the infiltration front (Spearman Rho test). PD-L1 and PD-1 positive staining at the centre of the tumour (c), the infiltration front (f), and simultaneous expression at the centre and front (cf). (**) *p* < 0.01, (***) *p* < 0.001.

| **CCRCC Tissue** | | **PD-L1c** | **PD-L1f** | **PD-L1cf** | **PD-1c** | **PD-1f** | **PD-1cf** |
| --- | --- | --- | --- | --- | --- | --- | --- |
| PD-L1c | r = | - | 0.429 ******* | 0.447 *** | 0.323 ** | 0.745 *** | 0.385 *** |
| PD-L1f | r = | 0.429 *** | - | 0.348 ** | 0.488 *** | 0.634 *** | 0.499 *** |
| PD-L1cf | r = | 0.447 *** | 0.348 *** | - | 0.573 *** | 0.348 ** | 0.742 *** |
| PD-1c | r = | 0.323 ** | 0.488 *** | 0.573 *** | - | 0.381 *** | 0.778 *** |
| PD-1f | r = | 0.745 *** | 0.634 *** | 0.348 ** | 0.381 ** | - | 0.369 *** |
| PD-1cf | r = | 0.385 *** | 0.499 *** | 0.742 *** | 0.778 *** | 0.369 *** | - |

**Table S3.** Systemic therapies received by 16 patients with metastatic CCRCC. Additional 7 patients with metastatic CCRCC did not receive systemic treatment.

| **Description of Systemic Treatment Used** | ***n* (%)** |
| --- | --- |
| Total number of patients receiving systemic therapy | 16 (100) |
| Patients treated with Tyrosine Kinase Inhibitors (TKIs) | 15 (93.75) |
| Patients treated with TKIs as first-line therapy | 14 (87.5) |
| Patients treated with TKIs as sequential therapy | 8 (50) |
| Patients treated with m-TOR inhibitors as first-line therapy | 1 (6.25) |
| Patients treated with m-TOR inhibitors as second-line therapy | 6 (37.5) |
| Patients treated with Immune Checkpoint Inhibitors (ICIs) as first-line | 0 (0) |
| Patients treated with ICIs as second-line | 8 (50) |
| Patients treated with combined ICIs | 0 (0) |

**Table S4.** Cox Regression model for 5-year overall survival (OS) prediction in CCRCC patients (Complete table with the first step and the final step of Wald method). Selected pathologic variables for analyses were: Fuhrman grade or G (low vs. high grade), tumour necrosis (no/yes), local invasion or pT (pT1 vs. pT2 vs. pT3-pT4), lymph node metastasis or N (no/yes) and distant metastases or M (no vs. synchronous vs. metachronous). Exponentiation of the B coefficient (ExpB) with confidence interval (CI) is also included. Statistically significant values are highlighted in bold. PD-L1c: combination of tissue and soluble isoforms of PD-L1.

| **A)** |  | **Tumour Centre** | | | | **Centre & Front** | | | | **Plasma** | | | |
| --- | --- | --- | --- | --- | --- | --- | --- | --- | --- | --- | --- | --- | --- |
| 5-year OS | **Variables** | ***p*** | **ExpB** | **CI** | | ***p*** | **ExpB** | **CI** | | ***p*** | **ExpB** | **CI** | |
| Multiple Cox Regression | Grade | 0.66 | 1.3 | 0.41 | 4.16 | 0.46 | 1.52 | 0.49 | 4.66 | 0.58 | 1.38 | 0.44 | 4.32 |
|  | Necrosis | 0.87 | 1.09 | 0.41 | 2.91 | 0.71 | 0.81 | 0.26 | 2.48 | 0.28 | 1.76 | 0.64 | 4.9 |
|  | pT | 0.08 | 1.8 | 0.93 | 3.49 | 0.11 | 1.67 | 0.9 | 3.1 | 0.04 | 1.9 | 1.02 | 3.5 |
|  | N | 0.03 | 4.06 | 1.11 | 14.9 | 0.005 | 5.59 | 1.66 | 18.8 | 0.39 | 1.82 | 0.47 | 7.05 |
|  | M | 0.01 | 1.99 | 1.18 | 3.36 | 0.008 | 2.02 | 1.2 | 3.39 | 0.001 | 2.62 | 1.49 | 4.61 |
|  | PD-L1 | 0.14 | 2.38 | 0.75 | 7.58 | 0.06 | 3.37 | 0.94 | 12.1 | 0.001 | 7.4 | 2.35 | 23.5 |
| Final Step of Wald Method | pT | **0.04** | 1.9 | 1 | 3.5 | **0.09** | 1.65 | 0.92 | 9 | **0.004** | 2.24 | 1.3 | 3.86 |
|  | N | **0.02** | 4.09 | 1.2 | 13.9 | **0.001** | 6.68 | 2.1 | 21.4 | - | | | |
|  | M | **0.01** | 2 | 1.19 | 3.38 | **0.005** | 2.07 | 1.24 | 3.46 | **8 × 10^−6^** | 2.83 | 1.68 | 4.75 |
|  | PD-L1 | **0.06** | 2.74 | 0.96 | 7.78 | **0.026** | 3.34 | 1.15 | 9.66 | **1 × 10^−5^** | 8.67 | 3.26 | 23.1 |
| **B)** | **Tumour Centre and Plasma** | | | | | **Font and Plasma** | | | | **Centre & Front and Plasma** | | | |
| 5-year OS | Variables | ***p*** | **ExpB** | **CI** | | ***p*** | **ExpB** | **CI** | | ***p*** | **ExpB** | **CI** | |
| Multiple Cox Regression | Grade | 0.53 | 1.42 | 0.48 | 4.24 | 0.22 | 1.97 | 0.66 | 5.84 | 0.28 | 1.81 | 0.62 | 5.3 |
|  | Necrosis | 0.95 | 0.97 | 0.36 | 2.63 | 0.57 | 0.74 | 0.26 | 2.08 | 0.49 | 0.69 | 0.24 | 1.96 |
|  | pT | 0.005 | 4.5 | 1.56 | 13.02 | 0.01 | 3.69 | 1.29 | 10.5 | 0.02 | 3.59 | 1.26 | 10.2 |
|  | N | 0.03 | 3.96 | 1.12 | 14.02 | 0.001 | 5.07 | 1.48 | 17.3 | 0.01 | 2.61 | 1.52 | 4.47 |
|  | M | 0.002 | 2.28 | 1.35 | 3.85 | 0.006 | 2.63 | 1.53 | 4.55 | 0.0001 | 8.8 | 2.16 | 35.8 |
|  | PD-L1c | 0.06 | 3.15 | 0.95 | 10.4 | 0.26 | 6.27 | 1.67 | 23.5 | 0.002 | 1.67 | 0.59 | 4.74 |
| Final Step of Wald Method | pT | **0.002** | 4.66 | 1.77 | 12.22 | **0.01** | 1.83 | 1.34 | 9.12 | **0.017** | 3.3 | 1.24 | 8.66 |
|  | N | **0.02** | 4.29 | 1.21 | 15.17 | **0.002** | 3.28 | 1.96 | 21.7 | **0.005** | 5.85 | 1.69 | 20.2 |
|  | M | **0.001** | 2.33 | 1.4 | 3.89 | **0.0001** | 5.13 | 1.6 | 4.5 | **0.0001** | 2.63 | 1.57 | 4.4 |
|  | PD-L1c | **0.03** | 3.5 | 1.09 | 11.28 | **0.009** | 2.56 | 1.53 | 19.7 | **0.003** | 7.98 | 2.05 | 31 |
